# Proteomic analyses bring new insights into the effect of a dark stress on lipid biosynthesis in *Phaeodactylum tricornutum*

**DOI:** 10.1038/srep25494

**Published:** 2016-05-05

**Authors:** Xiaocui Bai, Hao Song, Michel Lavoie, Kun Zhu, Yiyuan Su, Hanqi Ye, Si Chen, Zhengwei Fu, Haifeng Qian

**Affiliations:** 1 College of Environment, Zhejiang University of Technology, Hangzhou 310032, P. R. of China; 2 Department of Food Science and Technology, Zhejiang University of Technology, Hangzhou 310032, P. R. of China; 3 Quebec-Ocean and Takuvik Joint International Research Unit, Université Laval, Québec, Canada; 4 College of Biotechnology and Bioengineering, Zhejiang University of Technology, Hangzhou 310032, P. R. of China; 5 Xinjiang Key Laboratory of Environmental Pollution and Bioremediation, Chinese Academy of Sciences, Urumqi 830011, P. R. of China

## Abstract

Microalgae biosynthesize high amount of lipids and show high potential for renewable biodiesel production. However, the production cost of microalgae-derived biodiesel hampers large-scale biodiesel commercialization and new strategies for increasing lipid production efficiency from algae are urgently needed. Here we submitted the marine algae *Phaeodactylum tricornutum* to a 4-day dark stress, a condition increasing by 2.3-fold the total lipid cell quotas, and studied the cellular mechanisms leading to lipid accumulation using a combination of physiological, proteomic (iTRAQ) and genomic (qRT-PCR) approaches. Our results show that the expression of proteins in the biochemical pathways of glycolysis and the synthesis of fatty acids were induced in the dark, potentially using excess carbon and nitrogen produced from protein breakdown. Treatment of algae in the dark, which increased algal lipid cell quotas at low cost, combined with optimal growth treatment could help optimizing biodiesel production.

As the world demand of fossil fuels increases and the greenhouse gas carbon dioxide levels continues to rise, the development of cost-effective biofuels is of high importance and should be a priority^1^. Microalgae are considered as one of the most promising biodiesel sources because of their rapid growth rates and high lipid cell content^2^,^3^, i.e. more than 50% of dry weight in some nutrient-limited microalgal species^4^,^5^. Microalgae-derived biodiesel is currently viewed as an appealing alternative for fossil fuels since the scale-up of algal biofuel production could be sufficient to meet at least 5% of U.S. demand for transportation fuels^6^. In addition, domestic production of renewable fuels including algal biofuels has the potential to meet the dual goals of improving energy security and decreasing greenhouse gas emissions from the transportation sector^3^. However, the production cost of biodiesel is still too high hampering large-scale commercialization of algae-derived biodiesel and new efficient strategies are urgently needed to increase economic viability of biodiesel production.

Many studies have looked at the ecological and physiological factors regulating the production of lipids in autotrophic microalgae^1^,^3^,^7^. It is known that several environmental stressors (temperature, pH, salinity, and nutrient starvation) increase lipid cell quotas^8^,^9^. Nutrient starvation, nitrogen (N) especially proved to be one of the most successful approaches for enhancing lipid cell content in various microalgae species, such as *Chlorella emersonii*^10^(Trebouxiophyceae), *Parietochloris incise*^11^ (chlorophyceae), *Nannochloropsis spp*^12^ (Eustigmatophyceae), and *Phaeodactylum tricornutum*^7^,^13^,^14^ (Bacillariophyceae). Although N starvation and other environmental stressors decrease algal growth rate and total lipid production, it has been suggested that a combination of optimal growth conditions and growth-limited conditions could help improve biodiesel production^3^. Another strategy that has the potential to improve the cost-effectiveness of biofuel production would be to overexpress key genes of fatty acid synthesis via genetic engineering^14^. Even though the study of Niu *et al.*^15^ have shown that the overexpression of a type-2 diacylglycerol acyltransferase in a genetically engineered strain of a marine diatom can increase the rate of lipid biosynthesis, most studies failed to improve fatty acid biosynthesis rate in genetically modified algae^16^,^17^, indicating that the regulatory mechanisms of fatty acid synthesis are complex and poorly understood. However, downregulation of lipid catabolism pathways by genetic engineering could be a promising approach to increase lipid cell yield in eukaryotic microalgae without compromising growth as recently demonstrated in genetically engineered strains of the marine diatom *Thalassiosira pseudonana*^18^.

Diatoms are common unicellular eukaryotic algae that accounts for approximately 40% of global primary production^19^,^20^. Diatoms are particularly efficient to synthesize lipids compared to other microalgal species and show promise for potential future development of biodiesel production^6^. For instance, *P. tricornutum*, is one well-known marine diatom that can accumulate up to 61.4% of lipid per algal dry weight^5^. The use of marine diatoms for biofuel production is also advantageous because they can beneficiate of a vast unlimited supply of seawater as opposed to freshwater algae. Here we hypothesize that dark-treated *P. tricornutum* cells could preferentially utilize carbon (C) and nitrogen obtained from proteins breakdown to synthesize lipids. If true, this potential dark-induced lipid production would be useful for the optimization of biofuel production. In the present study, we submitted *P. tricornutum* to a long-term dark stress (4 d) and followed the cellular regulatory mechanisms leading to lipid accumulation in this species using a combination of physiological, proteomic (analysis of the algal proteome by isobaric tags for the relative and absolute quantitation, iTRAQ) and transcriptomic (qRT-PCR) approaches. The long-term dark stress inhibited several key proteins involved in nitrogen assimilation and in the synthesis of the photosynthetic machinery; simultaneously, key enzymes of glycolysis and the synthesis of fatty acids were induced apparently to assimilate the excess of C and N from protein breakdown. Moreover, an exposure for 1 to 4 days in the dark of algae at the end of exponential phase successfully increased lipid cell quotas relative to the light-exposed control cells.

## Results

### Effect of algal cell density and time on lipid and TAG production

To define the optimal initial cell density and exposure time, we tested in a preliminary experiment the effect of light on lipid production as a function of time and initial cell density (Treatment P1, P2 and P3). As shown in Table S1 and S2, the amount of neutral lipids and triacylglycerol (TAG) per cell decreased over time in the control (light) treatment irrespective of the initial cell density. However, in the dark treatment, the neutral lipid and TAG cell content increased by up to 2-fold during the first 4–5 days and then decreased for the remaining part of the 7-d experiment (see Supplementary Table S1 and S2). The total neutral lipid and TAG concentrations in the culture increased over time in the light treatment at all cell densities, whereas in the dark, their concentrations increased for the first 4–5 days (more than their respective light-exposed control) and then decreased until the end of the 7-d experiment (see Supplementary Table S3 and S4). Based on the above exploratory experiments, an initial cell density of 4 × 10^6^ (P2) cells/mL and an exposure time of 4 days were chosen for the subsequent experiments performed in the present study in order to maximize neutral lipid and TAG production. After the 4-day exposure (P2 treatment) in the light the cell density slightly increased by 20% whereas in the dark, the cell density slightly decreased by 17% (see Supplementary Fig. S1). Therefore, TAG or neutral lipid productivity (in g per liter of culture per day) was up to 50% higher after the 4 day exposure in the dark than in the light.

### Biochemical measurements and photosynthetic parameters

**Figure 1:**
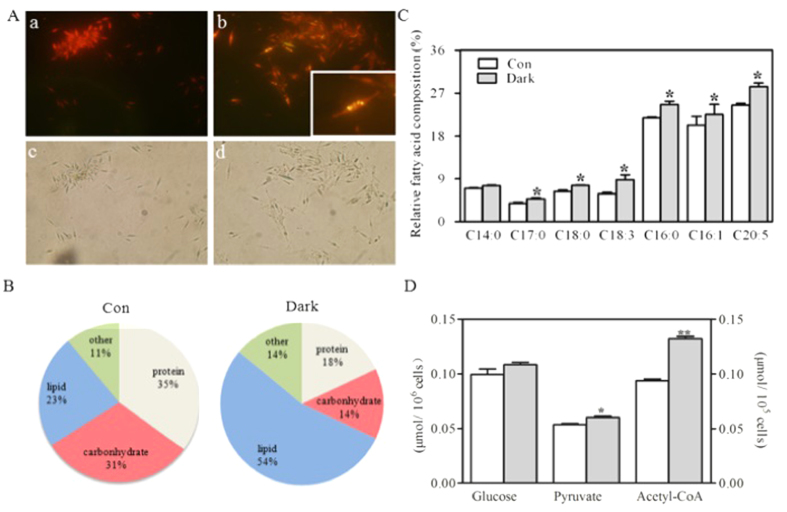
Total lipids, carbohydrates, and proteins as well as key metabolites of *Phaeodactylum tricornutum.* (**A**) Neutral lipid and chlorophyll fluorescence. Neutral lipid bodies were stained into yellow (a,b), red chlorophyll fluorescence observed in bright field (c,d), *P. tricornutum* grown in the light (a,c), *P. tricornutum* exposed to a 4-day dark stress (b,d). (**B**) Proportion of the algal dry weight incorporated in proteins, carbohydrates, lipids, and other minor compounds in *P. tricornutum* exposed for 4 days in the light (Con) or in the dark (dark). (**C**) Relative fatty acid composition (in percentage of total cellular fatty acids) of *P. tricornutum* exposed to optimal light (white bars, Con) and to a 4-day dark stress (black bars, Dark). (**D**) Cell content of glucose (μmol/10^6^ cells), pyruvate (μmol/ml 10^−6^), and acetyl-CoA (μmol/10^5^ cells) in *P. tricornutum* exposed for 4 d in the light (Con) or in the dark (Dark). Asterisks indicate significant according to independent Student’s t-tests (*P < 0.05, **P < 0.01). Values are mean ± SEM (n = 4).

When excited by blue light, nile red-stained *P. tricornutum* cells showed red fluorescence due to chlorophyll as well as yellow fluorescence due to neutral lipids (Fig. 1A). In Fig. 1A(a,b), we observed that yellow fluorescence intensities (indicative of neutral lipid accumulation in algal cultures) were stronger in the dark stressed-cells than in the light-exposed control cells. We then quantified algal lipid production, and found that total lipid per cell dry weight increased by 2.3-fold after 4 d of darkness compared with the control in the presence of light (Fig. 1B). Since the dry weight per cell and the cell volume did not change significantly for the cells harvested after the 4-d exposure in the light or in the dark (data not shown), it follows that the dark treatment also increased by 2.3-fold the total lipid amount per cell or per cell volume. Among the cellular lipids, the dominant fatty acids in *P. tricornutum* were tetradecanoate (C14:0), hexadecanoic acid (C16:0), 9-hexadecenoic acid (C16:1^ω9^), 7,10-hexadecadienoate (C17:0), octadecanoic acid (C18:0), linolenic acid (C18:3^ω9^), and cis-5,8,11,14,17-eicosapentaenoic acid (C20:5^ω3^). The proportion of six out of these seven fatty acids significantly increased in response to the 4-d dark stress relative to those of the control algae grown in the light (P < 0.05; Fig. 1C). When *P. tricornutum* cultures were grown in darkness, the proportion of each fatty acid relative to the total amount of fatty acid per cell increased significantly except for C14:0. By contrast, total carbohydrate and total protein per cell dry weight in the prolonged darkness group decreased by 48 and 55%, respectively (Fig. 1B). After the 4-d dark stress, the slight increase in the amount of glucose per cell was not statistically significant, but the amount of pyruvate and acetyl-CoA per cell increased significantly by around 1.1- and 1.4-fold (P < 0.05 and P < 0.001 for pyruvate and acetyl-CoA, respectively) relative to the control (Fig. 1D).

The content of chlorophyll a, chlorophyll c and carotenoid decreased in response to prolonged darkness by 67.2%, 49.7% and 66.9% relative to that of the control, respectively (see Supplementary Table S5). Also, the 4-d dark stress significantly decreased the Fv/Fm ratio and ETR by approximately 43% and 52%, respectively, relative to that of the control (see Supplementary Table S5).

### Effect of the dark treatment on cellular functions and biochemical pathways at the proteome level

**Figure 2:**
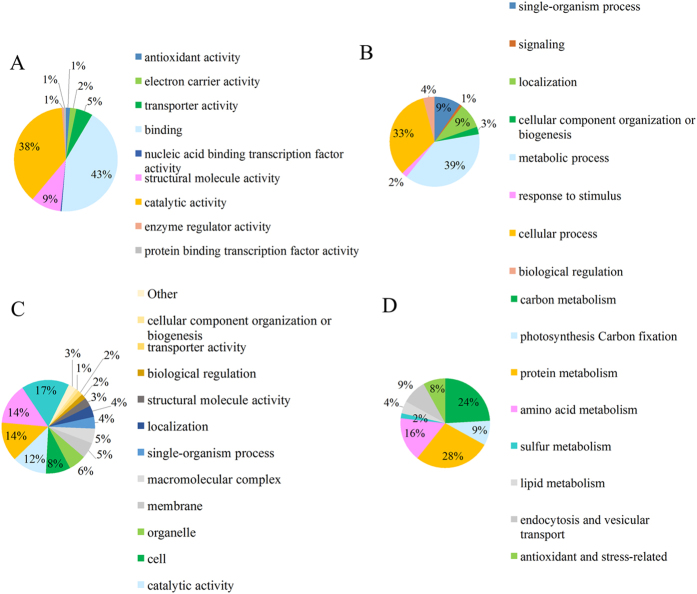
Classification of the differentially expressed proteins based on functional enrichment and KEGG pathway analysis. (**A**) The protein molecular functions, (**B**) the protein-associated biological processes, (**C**) the protein class, (**D**) and the KEGG biochemical pathways.

Using the 3-plex iTRAQ-based quantitative proteomic technology, 3,175 proteins were detected in *P. tricornutum*, among which 2,204 proteins were quantified. A ratio over 1.3 indicated up-regulation of protein expression while a ratio smaller than 1/1.3 (0.67) indicated down-regulation of protein expression. According to this criterion, 136 and 225 proteins were up- and down-regulated, respectively. All these differentially expressed proteins were represented on a metabolic pathway map (see Supplementary Fig. S2). The differentially expressed proteins were involved in numerous cellular functions based on functional enrichment and KEGG pathway analyses (Fig. 2). When classified on the basis of their molecular functions (Fig. 2A), the differentially expressed proteins involved in the binding of other cell constituents were the most abundant (43%) followed by proteins with catalytic activity (38%) and the remaining proteins were involved in various biochemical reactions in the cell. When considering the biological processes associated to each protein (Fig. 2B), 39% of the up- or down-regulated proteins were involved in metabolic processes, 33% in cellular process, and the remaining proteins were involved in several cell functions. When classified on the basis of protein class (Fig. 2C), most of the responsive proteins to the 4-d dark stress were involved in single-organism processes, cellular processes, and binding of intracellular components. The differentially expressed proteins based on KEGG metabolic pathways were mostly related to proteins, C, and amino acids metabolism (Fig. 2D). Among all the 361 up- or down-regulated proteins, only 53 proteins were assigned a functional annotation with KEEG pathway analysis, such as photosynthesis, C and energy metabolism, protein and amino acid metabolism, genetic information processing, glycolysis and fatty acid metabolism (see Supplementary Table S6).

### The darkness treatment affected photosynthetic pathway

During the prolonged darkness treatment, the expression of two photosystem I reaction center subunits (A0T0M6 and A0T0F3) decreased to 67.4% and 61.7% of that of the control, respectively. Moreover, the oxygen-evolving enhancer protein (B7FZ96) in PsbO and the CP47 chlorophyll apoprotein (A0T0B2) in PS II decreased to 50.3% and 35.7% of that of the control, respectively. The expression of two proteins related to fucoxanthin chlorophyll a/c binding proteins (B7G6Y1, B7FYL0) was down-regulated to 73.8% of the control. However, both glycine decarboxylase (B7FST3) and early light induced protein (B7FNY6) increased by 1.3-fold in response to the dark treatment.

### The darkness treatment affected carbon and energy metabolism

In response to the 4-d darkness treatment, the expressions of five proteins involved in the phosphopentose pathway were modulated. The expression of phosphoribulokinase (B5Y5F0), transaldolase (B5Y3S6), ribose 5-phosphate isomerase (B5Y3N7), and glyoxalase (B7GDI1) was significantly up-regulated while the expression of phosphoribosyltransferase (B7FST0) was down-regulated. The expression of isocitrate lyase (B7G518), an enzyme catalyzing the conversion of isocitrate into succinate within the glyoxylate cycle, increased by 1.34-fold whereas the expression of two ATP synthase subunits (A0T0E8 and A0T0F1) of the respiratory chain decreased by approximately 70% after the prolonged darkness treatment. The expression of UDP-glucose 6-dehydrogenase (B5Y5J6), involved in the biosynthesis of chrysolaminarin (a storage polysaccharide), decreased to 70.3% of that of the control. The abundance of proteins in the cytochrome family, such as the cytochrome b6-f complex iron-sulfur subunit (B5Y3C9), the cytochrome b6 (A0T0B8), and the cytochrome b559 subunit alpha (A0T0A3) also decreased significantly.

### The darkness treatment affected protein and amino acid metabolism

The expression of translation-related proteins in *P. tricornutum* strongly decreased after the darkness treatment. Indeed, five ribosomal proteins (B5Y502, B7FP80, A0T0C1, A0T0J1, and B7G0R5), two translation elongation factors (EFTs/EF1B and EFTu/EF1A) (Q9TK50 and B7GBQ5), and the translation initial factor 3 subunit A protein (B7G0T8) were all down-regulated by 61.7–73.9% relative to the expression level of the control. However, the abundance of factors involved in protein degradation increased. For instance, the expression of the ubiquitin-activating enzyme E1 (B7FTU2) increased by 1.4-fold after the dark treatment relative to that of the control. The abundance of proteins involved in the reduction of nitrate and nitrite into ammonium, such as nitrate reductase (B7G997) and glutamine synthetase (B7G5A1), decreased in the dark treatment by 40.4% and 74.1% compared to the expression level of the control, respectively, whereas the expression of the urea cycle-related proteins arginase (B7G627) and glutamate dehydrogenase (B7G3 × 3) increased by approximately 1.8- and 1.7-fold relative to the control group, respectively.

### The darkness treatment affected antioxidant and other stress-related proteins

The expression of Glutathione-S-transferase (B7FZ32) decreased by 61.8% in response to the dark stress. The abundance of proteins in a family of rhoassociated serine/threonine kinases (B7FR38, B7G086, B7GAH6 and B7FQ88) also decreased after the dark treatment by 45.4–67.8% relative to that in the control cultures. By contrast, the expression of two superoxide dismutase enzymes (B7FPQ3 and B7G0L6) significantly increased by 1.3- and 2.1-fold after the 4-d dark stress relative to the control group, respectively, and two heat shock proteins (B7FXQ8 and B5Y472) were dramatically upregulated in the dark treatment by more than 7-fold.

### The darkness treatment affected glycolysis and fatty acid metabolism

The abundance of two glyceraldehyde-3-phosphate dehydrogenases (B7G5Q1 and B7G6K6), phosphoenolpyruvate carboxykinase (B7GA05), and pyruvate kinase (B7G585) increased after 4 days of dark treatment by 1.3- to 1.5-fold relative to that of the control. The expression of one pyruvate dehydrogenase subunit (B7FXN2) increased by 1.7-fold in the dark treated-cells relative to that of the control, but the expression of the two other factors (B7GB47 and B7G3I7) of the pyruvate dehydrogenase complex rather decreased by more than 60% in the dark-treated cells relative to that of the control cultures. Regarding the biochemical pathways of fatty acid synthesis, the abundance of enoyl-acp reductase (FabI) and of the plastid lipid-associated protein in algal cells grown in prolonged darkness increased by 1.4- and 1.6-fold relative to that of the control, respectively, whereas the same dark treatment significantly decreased the expression of three proteins involved in fatty acid degradation (B7G529, B7FZ30 and B7FXX6) by 56.7 to 88.5% relative to that of the control.

### The effect of the dark treatment at the gene transcription level

**Figure 3:**
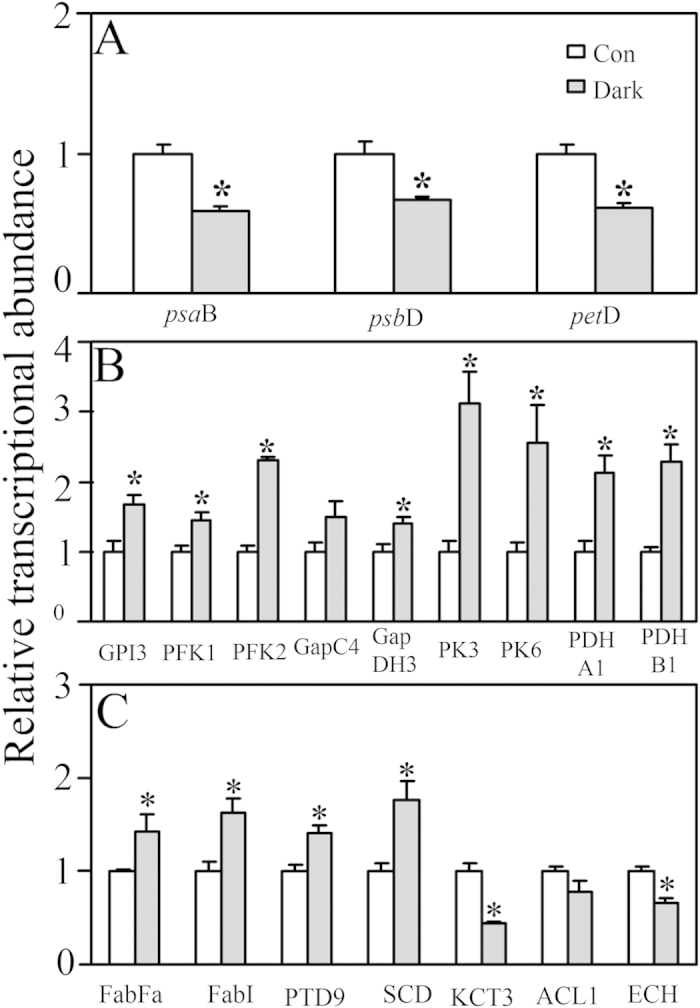
The transcription of key genes involved in *Phaeodactylum tricornutum* exposed for 4 days in the light (Con) or in the dark (dark). (**A**) Photosynthesis, (**B**) glycolysis, (**C**) fatty acid biosynthesis or oxidation. The transcription level of all genes was normalized to that of a housekeeping gene, actin rRNA. Asterisks indicate significant differences between both light treatments according to independent Student’s t-test (*P < 0.05, **P < 0.01). Values are mean ± SEM (n = 4).

The transcripts of *psb*D, *pet*D, and *psa*B in the dark-treated cells were down-regulated by 67.2%, 60.6% and 64.7% relative to those of the control group, respectively (Fig. 3A). The transcription of nine glycolysis-related genes significantly increased in response to the dark stress by 1.4- to 3.1-fold relative to that of the control (Fig. 3B). The transcripts of fatty acid synthesis genes (FabI, FabFa, SCD and PTD9) also increased significantly in the dark-treated cells relative to those in the control cells, whereas three β-oxidation genes (KCT3, ACL1 and ECH) decreased by 43.4 to 78.8% in the dark as shown in Fig. 3C.

## Discussion

**Figure 4:**
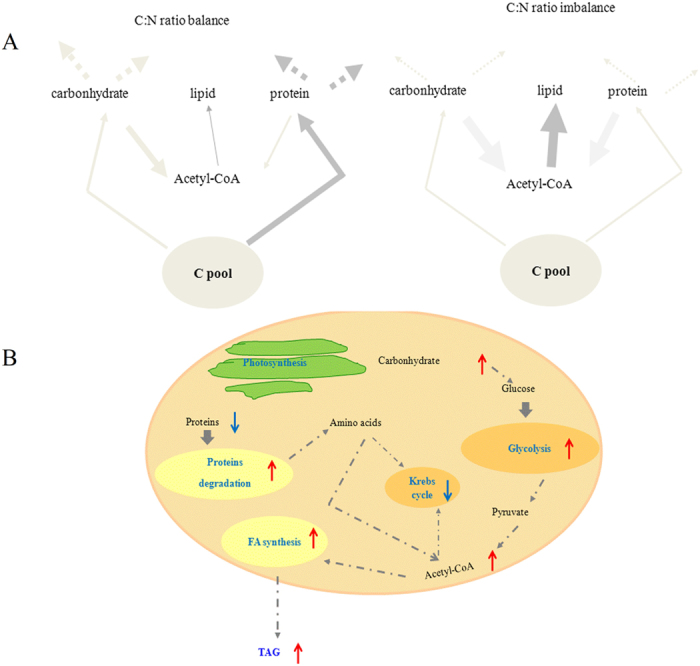
Integrated cell mechanisms leading to enhanced lipid production in the dark. (**A**) Metabolic fluxes of intermediate metabolites related to intermediate C and N metabolic pathways. The flux line width represents the relative magnitude of the flux. C:N ratio balance represents the control, C:N ratio imbalance represents the darkness treatment group. (**B**) Conceptual scheme of the dark-induced perturbation of fatty acid synthesis based on iTRAQ results.

The present study shows that total lipid cell quotas in *P. tricornutum*, one of the best strains for biodiesel production^5^, can strongly increase after a darkness treatment. When photosynthesis was abolished in the dark and synthesis of the proteins involved in the biosynthesis of the photosynthetic machinery was decreased, we found that cellular C, N and energy were redirected toward lipid biosynthesis (Fig. 4). Re-allocation of cellular energy toward lipid biosynthesis was also previously observed under N limitation and optimal light^14^,^21^. After a prolonged darkness period, the transcription and expression of key proteins involved in the production of the photosynthetic machinery (see Supplementary Table S6) as well as the biosynthesis of photosynthetic pigments (see Supplementary Table S5) only decreased by less than a factor of three even though C fixation was previously abolished for 4 d during the prolonged darkness period, a finding consistent with the results of Nymark *et al.*^22^. This suggests that a significant part of the photosynthetic apparatus, which contains a major fraction of cell N in algae, remains functional in the dark explaining why diatoms typically start to grow rapidly when the light suddenly becomes available after a dark stress^22^,^23^. Under such an intense stress on the photosynthetic apparatus, we indeed observed a decrease in the proportion of cell C allocated to carbohydrates and proteins, but carbohydrate oxidation through the glycolysis increased suggesting that *P. tricornutum* could either use an external source of sugars (such as sugars exuded by the algae in the culture medium during the growing period preceding the experiment in the dark) in the glycolysis pathway or mobilized polysaccharide reserves. The acetyl-CoA produced through the glycolysis was increasingly used for lipid biosynthesis. Our combined analyses of the whole proteome, key gene transcripts, organic acids, and enzymatic activities thoroughly describe the intricate cellular mechanisms at the root of the dark-enhanced lipid production in *P. tricornutum*.

### The long-term dark stress inhibited the synthesis of the photosynthetic machinery but stimulated carbohydrate oxidation

As previously observed by Qian and Borowitzka^24^ in N-limited *P. tricornutum* cells, the long-term dark stress we used in the present study decreased the cell carbohydrate content and affected the synthesis of the photosynthetic machinery, but stimulated lipid biosynthesis. The (nearly) generalized induction in the expression of glycolytic proteins (B7G5Q1, B7G6K6, B7GA05, and B7G585) and in the transcription of genes coding for glycolytic enzymes upon a prolonged dark stress clearly indicates that glucose conversion into acetyl-CoA was up-regulated. The expression of one enzyme (B7FXN2) of the pyruvate dehydrogenase complex (containing three enzymes: B7FXN2, B7GB47 and B7G3I7) was increased in response to the prolonged darkness period, but the expression of the two others were down-regulated. B7G317 (dihydrolipoamide acetyltransferase or E2) expression could decrease because it is an enzyme involved in the translation of *psb*A mRNA into the D1 protein in photosystem II (PSII)^25^, which was inhibited under dark stress. The cell content in pyruvate and acetyl-CoA effectively increased after the prolonged darkness period. However, the glucose cell content did not increase significantly in response to the dark treatment indicating that glucose was oxidized rapidly. The acetyl-CoA increasingly produced in the dark was then utilized for boosting fatty acid synthesis (see the section below). Furthermore, the dark-induced decrease in the expression of the enzyme UDP-glucose-6-dehydrogenase (B5Y5J6) suggested that the biosynthesis of polysaccharide reserves was inhibited, which could increase TAG biosynthesis^26^.

### Down regulation of nitrogen assimilation under long-term darkness

The decrease of two key enzymes of N assimilation in darkness, nitrate reductase and glutamine synthetase, suggested that N assimilation into amino acids and proteins decreased under the prolonged dark stress. A decrease in the protein cell content was indeed observed after the 4-d dark stress. Furthermore, the decrease in the expression of five ribosomal proteins and two translation elongation factors in the dark is also consistent with a dark-induced inhibition of protein biosynthesis (Fig. 1B). An inhibition of the N assimilatory pathway in response to the prolonged darkness period contrasted with the up-regulation in the expression of the enzymes involved in N assimilation observed under N limitation in *P. tricornutum*^14^. This suggests that after a prolonged period of time in the dark, *P. tricornutum*, decrease energy investment in the N assimilation biochemical machinery by contrast to the observed up-regulation in the expression of the enzymes involved in N assimilation under N limitation in *P. tricornutum*^14^. However, both N limitation/starvation and a prolonged dark stress inhibit net protein biosynthesis. For instance, the transcripts level of two ribosomal proteins (ProtIDs 21235 and 15259) and of a translation factor (ProtID 269148) decreased in *T. pseudonana* grown under N starvation^27^.

We found that the dark stress not only decreased protein biosynthesis, but it also increased protein breakdown by inducing the expression of an ubiquitin-activating enzyme E1 (B7FTU2), which catalyzed the first step in protein degradation process^28^,^29^. *P. tricornutum* cells redirect the acetyl-CoA produced from glycolysis toward lipid production, while concurrently, inhibiting the allocation of carbon skeleton to the nitrogen assimilation machinery (Fig. 4).

### Oxidative stress increased in prolonged darkness

Imbalance in C and N assimilation in algal cells can lead to oxidative stress^30^. In the present study, the observed increase in the expression of two superoxide dismutase isozymes (B7FPQ3 and B7G0L6) and heat-shock proteins (B7FXQ8 and B5Y472) after the 4-d dark stress suggests that the production of reactive oxygen species (ROS) in dark-treated cells increased and that reparation of proteins damaged by ROS was increasingly needed. Moreover, the increase in fatty acid synthesis measured in the dark-stressed cells could serve not only as an energy store, but also as antioxidant as suggested by Li *et al.*^31^. The later authors argued that TAG incorporation in thylakoids membranes could alleviate ROS accumulation by blocking redundant electrons passing through the photosynthetic electron transport chain.

### Stimulation of fatty acid synthesis under a long-term dark stress

The first step in fatty acid synthesis is the conversion of acetyl-CoA to malonyl-CoA, catalyzed by acetyl-CoA carboxylase (ACCase). Even though the dark treatment did not increase the expression of the ACCase of *P. tricornutum*, it did increase the expression of the enzyme enoyl-acp reductase (FabI), which is the key enzyme to elongate fatty acid carbon chains, hence facilitating the synthesis of long-chain fatty acids. Real-time quantitative PCR also demonstrated the significant increase in the transcription of a number of genes involved in fatty acid biosynthesis (i.e. FABI, FABFa, SCD and PTD9) under prolonged darkness.

Furthermore, the expression and gene transcription of a number of enzymes involved in the β-oxidation of fatty acids was down-regulated in response to the 4-d dark stress suggesting that fatty acid degradation decreased in the dark relative to the control. The results suggest that oxidation of lipids was not a major energy and C source for *P. tricornutum* in the dark. Hence, the observed decrease in the expression of enzymes involved in fatty acid β-oxidation and the demonstrated increase in the expression of several enzymes of fatty acid biosynthesis in the dark-treated cells act together to increase the TAG cell content in the dark-treated cells. By contrast, it has been shown that the increase in TAG production by N-limited *P. tricornutum* cells is coupled to an increased allocation of C and reductant to lipid biosynthesis rather than to an increase in the activity of the enzymes involved in fatty acid biosynthesis^14^.

In the dark-treated cells, we observed that hexadecanoic acid (C16:0) and octadecanoic acid (C18:0) production increased to high level; both fatty acids can be esterified to acyl carrier protein (ACP), 16:0-ACP and 18:3-ACP, and these two ACPs fatty acids may contribute to the biosynthesis of glycerolipids in the chloroplast and thylakoid membranes^32^. Chloroplast membranes regulate metabolites in and out of cells, and thylakoid membranes are the site of the primary process of photosynthesis. Interestingly, the dark treatment also increased the production of long chains unsaturated fatty acids (e.g. linolenic acid and cis-5,8,11,14,17-eicosapentaenoic acid or EPA) by inhibiting fatty acid β-oxidation and increasing the expression of the enzyme enoyl-acp reductase (FabI). These long chains unsaturated fatty acids are of high nutritional values for farmed animals as well as humans^33^. A dark treatment of *P. tricornutum* thus needs to be considered in future development of algal cultivation technique in aquaculture and health food industries.

### Fluxes in carbon and nitrogen assimilatory pathways and regulation of lipid biosynthesis

Our comprehensive physiological, gene transcripts, and proteomic analyses show that a long-term dark stress stimulated glycolysis, protein degradation, and fatty acid synthesis explaining the observed increase in lipid cell content in the dark (Fig. 4). Under long-term darkness, up-regulation of the glycolysis pathway and the associated increase in the production of NADPH, pyruvate, and acetyl-CoA all contribute to increase lipid biosynthesis whereas, under N limitation, redundant photosynthetically fixed C and NADPH enhanced fatty acid and TAG biosynthesis^31^,^34^. The flux of C toward protein synthesis in the marine alga *P. tricornutum* tended to dominate in optimal growth conditions (balance C:N ratio) (Fig. 4A), while under a long-term dark stress, cell C and N from protein breakdown tended to be redirected toward lipid biosynthesis to minimize the imbalance in C and N assimilation (Fig. 4A).

We demonstrated that a long-term dark stress in the marine microalga *P. tricornutum* enhanced neutral lipid cell content by 3.7-fold without strongly decreasing cell biomass by redirecting C and N flux into lipid synthesis (see Supplementary Table S1). This increase in lipid cell content after 4 day in darkness is higher than the increase in lipid cell quotas measured in several microalgae species growing in conditions of low N concentrations^3^,^35^. This storage of high-energy lipids could be of paramount importance for the survival of *P. tricornutum* in conditions of prolonged darkness such as in the deep ocean.

Our study brings new insights into the fundamental mechanisms leading to modulation of lipid production in the dark in *P. tricornutum*, a species with high potential for biofuel production. Dark treatment could strongly stimulate lipid cell quotas, and represented a new cheap way to enhance algal lipid cell quotas. If combined with optimal culture conditions, a dark stress could potentially increase lipid productivity for biofuel production.

## Methods

### Culture conditions

The axenic microalga *P. tricornutum* (Strain 863) was obtained from the Institute of Hydrobiology of the Chinese Academy of Sciences. The algae cultures were maintained in sterilized Erdschreiber’s medium (without soil extract) under cool white fluorescent tubes at an irradiance of 54 μmol m^−2^ s^−1^) and a 12 h light:12 h dark cycle. The temperature was kept at 22 ± 0.5 °C. The cell density was estimated spectrophotometrically by following the optical density at 680 nm (OD_680_) using a calibration curve of cell density (measured in a control diatom culture with a hemocytometer) as a function of OD_680_. The following regression equation was used for predicting cell density (y in ×10^5^ cells/mL) from OD_680_ (x): y = 100x + 1.12 (R^2^ = 99.08)^36^. Algae were inoculated at an initial cell density of 4 × 10^6^ cells/mL and grown in the conditions described above until the end of exponential growth phase, i.e. for 11 (treatment P1; 2 × 10^7^ cells/mL), 17 (treatment P2; 4 × 10^7^ cells/mL), and 27 (treatment P3; 8 × 10^7^ cells/mL) days. For each treatment (P1, P2, and P3), cultures were subsequently grown in the dark for 2 to 7 additional days as they gradually enter in the stationary growth phase whereas other cultures were grown in the light for the same period of time in parallel. Measurements of total lipid concentrations and cell quotas in triacylglycerol (TAG) and neutral lipid (see sections below for details on the lipid analyses) were performed after 2 to 7 days of growth in the dark or in the light while all other biochemical, transcriptomic and proteomic measurements were performed after 4 days of growth (and for the treatment P2 only) in the dark or in the light. Data are representative of three independent experiments with four replicates performed for each measurement.

### Analysis of algal pigments and *in vivo* photosynthetic parameters

Aliquots of 10 ml from each culture were centrifuged to extract pigments from *P. tricornutum*. The pellets were soaked in 5 mL acetone (90%, v/v) and stored at 4 °C for 24 h in the dark. Spectrophotometry was then used to measure the algal content of chlorophyll a (Chl a), chlorophyll c (Chl c) and carotenoids according to the method of Jeffrey *et al.*^37^. Chlorophyll fluorescence was determined *in vivo* using MINI-PAM- II system (Heinz Walz GmbH, 910090 Effeltrich, Germany); The Fv/Fm ratio, effective photochemical efficiency of photosystem II (PSII), and electron transport rate (ETR) were analyzed. Cultures were dark acclimated for 15 to 20 min before *in vivo* chlorophyll fluorescence determination.

### Lipid and fatty acid profile analysis

The detection and quantification of neutral lipids in algae were done according to the method of Chen *et al.*^38^, which used the nile red fluorescence dye. TAG kits (Nanjing Jiancheng Bioengineering Institute, Nanjing, China) were used to determine the TAG cell content. Total fatty acid concentrations in algal cultures were analyzed by gas chromatography directly from algal cultures as previously reported by Work *et al.*^39^. Briefly, 1.0 mL of 1 M NaOH in 95% methanol was added to 0.5 mL of algae culture and then heated in tightly sealed vials at 100 °C for 2 h to induce cell lysis and saponify lipid. Acid-catalyzed methylation was accomplished by adding 1.5 mL of 12 N HCl: methanol (1:16, v/v) and incubating at 80 °C for 5 h. Fatty acid methyl esters were extracted into 1.25 mL of hexane through gentle inversions for 20 min. Extracts were washed with distilled water and analyzed on the BD-INNOWax gas chromatograph equipped with a capillary column (30 m × 0.25 mm i.d.) with 0.2-mm film thickness and a flame ionization detector at 220 °C.

### Analysis of total carbohydrate, protein, glucose, pyruvate and acetyl-CoA

Total carbohydrate was determined according to Mauro *et al.*^40^. In brief, phenol (1.0 mL, 5% w/w) and H_2_SO_4_ (5.0 mL, 95 ~ 98% v/v) were added to 2.0 mL of microalgae cultures. The mixtures were shaked 30 min at ~25–30 °C for reaction, and then light absorption was measured at 485 nm. The total content of protein was measured using a protein assay kit (Beyotime, Jiangsu, China). The glucose, pyruvate and acetyl-CoA cell content was determined using a commercially available glucose, pyruvate and acetyl-CoA kits (Shanghai Rongsheng Bioengineering Company, Shanghai; Nanjing Jiancheng Bioengineering Institute, Jiangsu; Comin, Suzhou, China, respectively), respectively, according to the instruction provided by the manufacturer.

### Total protein extraction and iTRAQ analysis

Crude proteins from *P. tricornutum* were extracted and digested with trypsin (Promega) at an enzyme-to-substrate ratio of 1:50 for 12 h at 37 °C. Equal amounts of iTRAQ labeled (AB Science) peptides from each group were mixed and resolved into 15 fractions by HPLC, followed by Q Exactive mass spectrometry (Thermo Fisher Scientific). The resulting MS/MS data were searched against a Uniprot *P. tricornutum* protein database using MaxQuant (v1.0.13.13) with false discovery rate of 1%. A functional annotation was attributed to the quantified proteins using gene ontology (GO) annotation, Kyoto Encyclopedia of Genes and Genomes (KEGG) pathway analysis, functional enrichment and secondary structure analysis. Two technical replicates with tag swapping were also conducted for each biological replicate.

### Gene transcription analysis

qRT-PCR was used to quantify the transcript levels of key genes involved in photosynthesis, glycolysis and fatty acid biosynthesis and degradation. Three photosynthesis-related genes, *psb*D, *pet*D, and *psa*B, encoding key proteins in PS II, Cyt b6f, and PS I, respectively, were selected. The nine selected glycolysis-related genes were glucose-6-phosphate isomerase 3 (GPI3), glyceraldehyde-3-phosphate dehydrogenase C4 (GapC4), glyceraldehyde-3-phosphate dehydrogenase (GapDH3), 6-phosphofructokinase (PFK1 and PFK3), pyruvate kinase (PK3 and PK6), pyruvate dehydrogenase E1 component alpha (PDHA1) and pyruvate dehydrogenase E1 component beta subunit (PDHB1). The four selected fatty acid biosynthesis genes were Enoyl-[acyl-carrier-protein] reductase (using NADH) (FabI), 3-oxoacyl-[acyl-carrier-protein] synthase (FabFa), short-chain dehydrogenase/reductase (SCD), and delta 9 desaturase (PTD9). Finally, the four selected fatty acid β-oxidation genes were 3-ketoacyl-CoA thiolase (KCT3), acyl-CoA dehydrogenase (ACD), acyl-CoA ligase 1 (ACL1), and enyol- CoA hydratase (ECH). Total RNA was extracted from diatom cells using RNAiso (Takara Company, Dalian, China) according to the manufacturer’s instructions. RNA was reverse-transcribed into cDNA using a reverse transcriptase kit (Toyobo, Tokyo, Japan). Real-time quantitative PCR was then performed using an Eppendorf Master Cycler® ep RealPlex^4^ (Wesseling-Berzdorf, Germany) and SYBR Green PCR reagents (Toyobo, Tokyo, Japan). The following two-step PCR protocol was used: cDNA denaturation at 95 °C for 1 min followed by 40 cycles of denaturation (95 °C for 15 s) and annealing (60 °C for 1 min). To eliminate variations in the quantity and quality of mRNA and cDNA, the results were normalized to the transcription level of a housekeeping gene, 18S rDNA. The relative level of gene transcription among the treatment groups was quantified using the 2^−ΔΔCt^ method^41^.

### Data analysis

The data are presented as the mean ± standard error of the mean (SEM). Significant statistical differences among means were evaluated using one-way analyses of variance (ANOVA). Values were considered significantly different when the probability (p) was <0.05. All statistical analysis was performed using the StatView 5.0 program (Statistical Analysis Systems Institute, Cary, NC).

## Additional Information

**How to cite this article**: Bai, X. *et al.* Proteomic analyses bring new insights into the effect of a dark stress on lipid biosynthesis in *Phaeodactylum tricornutum. Sci. Rep.* **6**, 25494; doi: 10.1038/srep25494 (2016).

## Supplementary Material

Supplementary Information
