# Supplementary material for: Proteomic analyses bring new insights into the effect of a dark stress on lipid biosynthesis in Phaeodactylum tricornutum
Source: Sci Rep. 2016 May 5;6:25494. doi: 10.1038/srep25494 (PMC4857112; doi:10.1038/srep25494)
Supplement: Supplementary Information [file srep25494-s1.pdf]

Supporting information for

Proteomic analyses bring new insights into the effect of a dark stress on lipid biosynthesis in *Phaeodactylum tricornutum*

Xiaocui Bai[1,2], Hao Song[2], Michel Lavoie[3], Kun Zhu[4], Yiyuan Su[2], Hanqi Ye[4], Si Chen[2], Zhengwei Fu[4], Haifeng Qian[1,5,*]

1 College of Environment, Zhejiang University of Technology, Hangzhou 310032, P. R. of China

2 Department of Food Science and Technology, Zhejiang University of Technology, Hangzhou 310032, P. R. of China

3 Quebec-Ocean and Takuvik Joint International Research Unit, Université Laval, Québec, Canada

4 College of Biotechnology and Bioengineering, Zhejiang University of Technology, Hangzhou 310032, P. R. of China

5 Xinjiang Key Laboratory of Environmental Pollution and Bioremediation, Chinese Academy of Sciences, Urumqi 830011, P. R. of China

***Corresponding author:**

Haifeng Qian

College of Environment, Zhejiang University of Technology, Hangzhou 310032, P. R. of China. E-mail: hfqian@zjut.edu.cn

Table S1. Neutral lipid cell quotas as a function of culture time and cell density (P1, P2, P3) for the optimal light and dark treatment. Nile Red Fluorescence intensity (a.u.) (/ 10^7 cells).

Cell density	P1		P2		P3	
time	Con	Dark	Con	Dark	Con	Dark
2d	10	16.2	6.5	11.5	5.9	7.7
3d	9.5	21.5	6.3	15	5.7	7.9
4d	8.4	24.1	5.6	20.9	5.5	9
5d	7.2	24.6	5.4	20.2	5.4	9.3
6d	6.9	23.9	5.3	20.4	5.4	9.5
7d	5.9	23.6	5.4	20.8	5.3	9.4

Table S2. TAG Content per cell (nmol TAG/10^7 cells) as a function of culture time, cell density (P1, P2, and P3) for the optimal light and dark treatment.

Cell density	P1		P2		P3	
time	Con	Dark	Con	Dark	Con	Dark
2d	0.93	1.89	0.58	0.76	0.28	0.44
3d	0.89	2.03	0.55	0.96	0.27	0.51
4d	0.78	2.04	0.49	1.82	0.26	0.72
5d	0.66	2.12	0.45	1.81	0.28	0.69
6d	0.59	2	0.41	1.77	0.29	0.71
7d	0.52	1.92	0.35	1.77	0.24	0.72

Table S4. Total yield of TAG in one liter of algal culture (nmol L-1) as a function of culture time, cell density (P1, P2, and P3) for the optimal light and dark treatment.

Table S3. Total yield of neutral lipid (Nile Red Fluorescence intensity) in 1L cultural medium as a function of culture time, cell density (P1, P2, and P3) for the optimal light and dark treatment.

Cell density	P1		P2		P3	
time	Con	Dark	Con	Dark	Con	Dark
2d	2452	2935	2848	3705	5128	5553
3d	2671	3874	2906	4593	5103	5500
4d	2698	4198	2891	6295	5155	6086
5d	2673	4236	3003	6003	5325	6149
6d	2837	4044	3239	5940	5595	6215
7d	3016	3946	3894	6036	5889	6074

Table S4. Total yield of TAG in one liter of algal culture (nmol L^{-1}) as a function of culture time, cell density (P1, P2, and P3) for the optimal light and dark treatment.

Cell density	P1		P2		P3	
time	Con	Dark	Con	Dark	Con	Dark
2d	228.04	342.47	254.16	244.87	243.38	317.33
3d	250.27	365.81	253.66	293.95	241.70	355.06
4d	250.54	355.37	252.94	548.18	243.67	486.86
5d	244.99	365.06	250.29	537.93	276.14	456.23
6d	242.61	338.40	250.59	515.42	300.50	464.48
7d	265.82	321.02	252.42	513.65	266.69	465.26

Table S5. Cell quotas of chlorophyll a, chlorophyll c, and carotenoids as well as Fv/Fm ratio and electron transport rate (ETR) measured in *P. tricornutum* exposed for 4 days either in the light (Con) or in the dark (D). Asterisks indicate significant differences determined by an independent Student's-t-test (*P<0.05, **P<0.01).

	Chla (pg $cell^{-1}$)	Chl c (pg $cell^{-1}$)	Carotenoid (pg $cell^{-1}$)	Fv/Fm (ratio)	ETR (μmol electrons $m^{-2}s^{-1}$)
Con	27.18	4.28	15.48	0.529	20.65
Dark	18.26**	2.13**	10.35**	0.165*	10.78*

* Represents a statistically significant difference relative to the control (*, $p < 0.05$; **, $p < 0.01$).

Table S6. Proteins (and their assigned functional annotation) differentially expressed in *Phaeodactylum tricornutum* upon a 4-day dark stress. Ratio represents the protein expression level in the dark normalized to that in the light.

Spot No.	Protein description	Ratio
Photosynthesis		
A0T0M6	photosystem I reaction center subunit XI	0.6735
A0T0F3	Photosystem I reaction center subunit IV	0.617
B7FZ96	Oxygen-evolving enhancer protein 1	0.503
A0T0B2	Photosystem II CP47 chlorophyll apoprotei	0.3565
B7FYL0	Protein fucoxanthin chlorophyll a/c protein	0.7485
Carbon and energy metabolism		
B5Y5F0	Phosphoribulokinase	1.522
B7GDI1	Glyoxalase	1.389
B5Y3S6	Transaldolase	1.629
B5Y3N7	Ribose 5-phosphate isomerase	1.5095
B7FST0	ATP phosphoribosyltransferase	0.608
B7G518	Isocitrate lyase	1.3355
B7FST3	Glycine decarboxylase	1.3055
B5Y3C9	Cytochrome b6-f complex iron-sulfur subunit	0.6005
A0T0B8	Cytochrome b6	0.742
A0T0A3	Cytochrome b559 subunit alpha	0.725
A0T0E8	ATP synthase subunit b, chloroplastic	0.701
A0T0F1	ATP synthase subunit alpha, chloroplastic	0.768
Protein metabolism and Amino acid metabolism		
B7G0T8	Eukaryotic translation initiation factor 3 subunit A	0.7385
B5Y502	Ribosomal protein L15	0.6605
B7FP80	40S ribosomal protein S8	0.644

A0T0C1	50S ribosomal protein L1, chloroplastic	0.6165
A0T0J1	50S ribosomal protein L5, chloroplastic	0.713
B7G0R5	60S ribosomal protein L18a	0.674
Q9TK50	Elongation factor Ts, chloroplastic	0.715
B7GBQ5	Translation elongation factor EFTu/EF1A	0.688
B7FTU2	Ubiquitin-activating enzyme E1, protein 3	1.434
B7G997	Nitrate reductase	0.404
B7G5A1	Glutamine synthetase	0.741
B7G627	Arginase	1.783
B7FT50	Asparagine synthetase	1.3745
B7G3X3	Glutamate dehydrogenase	1.702
Glycolysis and fatty acid metabolism		
B7G5Q1	Glyceraldehyde-3-phosphate dehydrogenase	1.3985
B7G6K6	Glyceraldehyde-3-phosphate dehydrogenase	1.513
B5Y5J6	UDP-glucose 6-dehydrogenase	0.7025
B7GA05	Phosphoenolpyruvate carboxykinase	1.51
B7G585	Pyruvate/Phosphoenolpyruvate kinase	1.308
B7FXN2	FAD-linked oxidoreductase	1.711
B7GB47	Methylenetetrahydrofolate reductase	0.695
B7G3I7	Dihydrolipoamide acetyl transferase	0.6145
B7FS72	Enoyl-acp reductase	1.463
B7GC65	Plastid lipid-associated protein	1.609
B7G529	Inorganic pyrophosphatase	0.567
B7FZ30	Enoyl-coa hydratase	0.885
B7FXX6	Long chain acyl-coa synthetase	0.6995
Antioxidant and other stress-related proteins		
B7FPQ3	Superoxide dismutase	1.354
B7G0L6	Cyto superoxide dismutase	2.127
B7FZ32	Glutathione S-transferase	0.6175

B7FR38	Serine/threonine-protein kinase	0.454
B7G086	Serine/threonine-protein kinase	0.5415
B7GAH6	Serine/threonine-protein kinase	0.5475
B7FQ88	Serine/threonine-protein kinase	0.678
B7FXQ8	Heat shock protein 20	7.237
B5Y472	Heat shock protein 20	7.684

Figure Legend

Figure S1. *P. tricornutum* cell density after dark treatment.

Figure S2. *P. tricornutum* metabolic pathway map. Light grey background traces indicate KEGG pathways that not are expressed in the proteome of *P. tricornutum*. Proteins that are up- or down-regulated during dark stress are labeled in red and green, respectively. (FA, fatty acid)

Figure S1

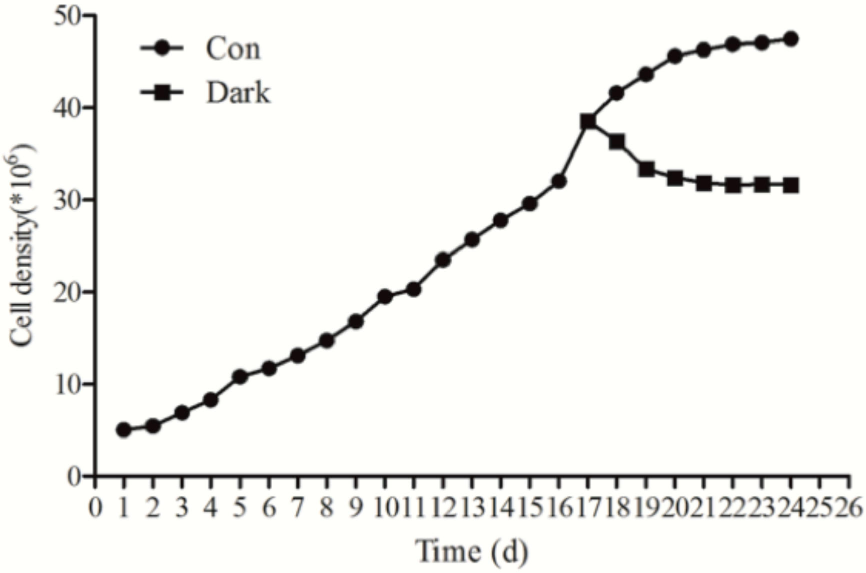

Con
Dark
Cell density(*10^6)
50
40
30
20
10
0
0 1 2 3 4 5 6 7 8 9 10 11 12 13 14 15 16 17 18 19 20 21 22 23 24 25 26
Time (d)

Figure S2

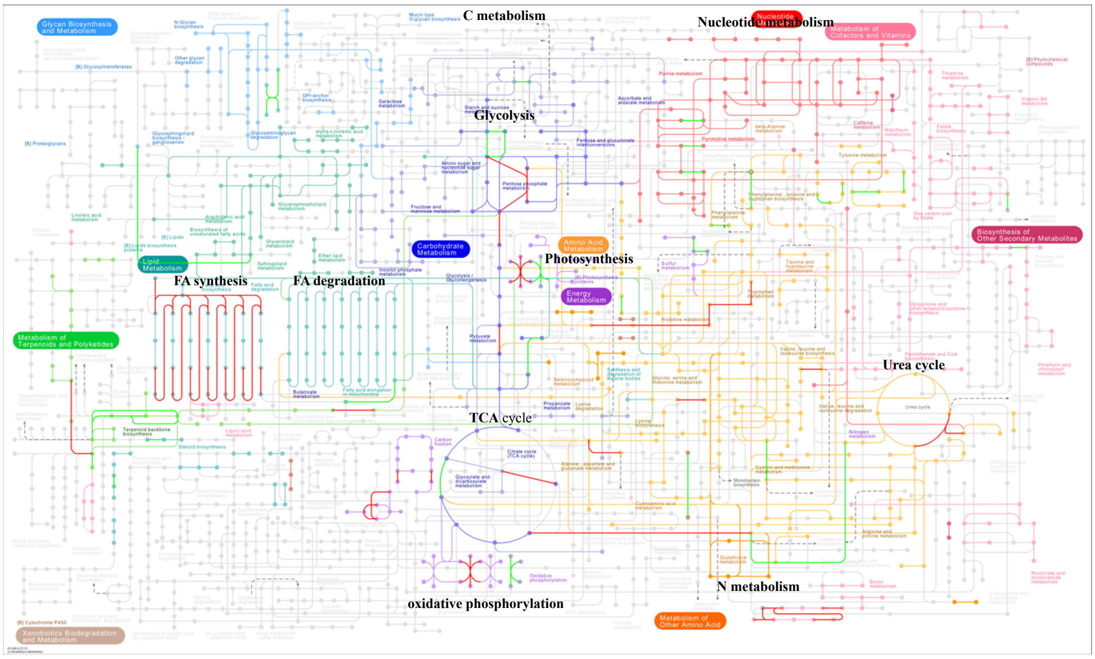

C metabolism
Nucleotide metabolism
Glycan Biosynthesis and Metabolism
Metabolism of Cofactors and Vitamins
Glycolysis
Biosynthesis of Other Secondary Metabolites
Carbohydrate Metabolism
Amino Acid Metabolism
Photosynthesis
Lipid Metabolism
FA synthesis
FA degradation
Energy Metabolism
Metabolism of Terpenoids and Polyketides
Urea cycle
TCA cycle
N metabolism
oxidative phosphorylation
Metabolism of Other Amino Acid
Xenobiotics Biodegradation and Metabolism
